# Research on the Evaluation of Moral Education Effectiveness and Student Behavior in Universities under the Environment of Big Data

**DOI:** 10.1155/2022/2832661

**Published:** 2022-07-30

**Authors:** Rui Zhu

**Affiliations:** Publicity Department, Shandong Management University, Jinan, Shandong 250000, China

## Abstract

Traditional moral evaluation relies on artificial and subjective evaluation by teachers, and there are subjective errors or prejudices. To achieve further objective evaluation, students' classroom performance can be identified, and the effectiveness of moral education can be evaluated based on student behavior. Since student classroom behavior is random and uncertain, in order to accurately evaluate its indicators, a large amount of student classroom behavior data must be used as the basis for analysis, while certain techniques are used to filter out valuable information from it. In this paper, an improved graph convolutional network algorithm is proposed to study students' behaviors in order to further improve the accuracy of moral education evaluation in universities. The technique of video recognition is used to achieve student behavior recognition, thus helping to improve the quality of moral education evaluation in colleges and universities. First, the multi-information flow data related to nodes and skeletons are fused to improve the computing speed by reducing the number of network parameters. Second, the spatiotemporal attention module based on nonlocal operations is constructed to focus on the most action discriminative nodes and improve the recognition accuracy by reducing redundant information. Then, the spatiotemporal feature extraction module is constructed to obtain the spatiotemporal association information of the nodes of interest. Finally, the action recognition is realized by the Softmax layer. The experimental results show that the algorithm of action recognition in this paper is more accurate and can better help moral evaluation.

## 1. Introduction

Moral evaluation is a guide and an initiative to carry out moral education in schools. Moral evaluation is defined in the Dictionary of Education as “the process of making value judgments on the performance of moral behavior of individuals using the acquired moral standards” [1]. The broad perspective of school moral evaluation content is to examine the ideological, moral, and political qualities of individuals, and the narrow perspective is to examine the moral qualities of individuals. Both focus on the moral cognition and moral behavior of individuals, especially the moral behavior that is more easily observed [2].

To adhere to “people-oriented” means to maintain human dignity, respect human rights, give full play to human potential, meet human needs, and promote the all-round development of people. By insisting on the college students as the center, we should not only educate them, guide them, inspire them and spur them on but also respect them, understand them, care for them, and help them to develop good ideological and moral qualities and excellent ideological and political qualities, so as to achieve the purpose of moral education and realize the fundamental goal of establishing moral education for people [3].

In the current reform of quality education, colleges and universities pay more and more attention to the moral education quality cultivation of students, and no longer focus not only on the teaching of students' professional courses but also begin to focus on the overall development of students' comprehensive quality. In order to implement the requirements of quality education cultivation and promote the vigorous development of moral quality education, a matching moral quality evaluation system for college students should be formulated. The traditional evaluation method of students' moral quality has been rather backward, and it is difficult to ensure the fairness and scientificity by simply relying on human for evaluation, which does not meet the needs of quality education, so it needs technology updating. Innovate student moral quality evaluation mode, introduce information technology support, and use big data technology and computer information technology to create a sound moral quality evaluation system for college students. Scientific design and optimization of system structure in order to improve the efficiency of moral quality education in colleges and universities and guarantee the quality of moral quality education for college students.

Literature [4] constructed the spatiotemporal graph with the natural connections of human joints and proposed the spatiotemporal network model with the graph convolution layer as the basic module. Literature [5] integrated a discrete multiscale aggregation scheme and the spatiotemporal graph convolution operator called G3D to form a powerful feature extraction structure. Literature [6] introduces a context-encoded network for enhancing contextual feature relevance and automatically learning the skeleton topology. Literature [7] incorporates third-order features to effectively capture the relationship between joints and body parts. Literature [8] introduced a novel progressive multiscale convolution for capturing long- and short-term correlations in the spatial and temporal domains. Literature [9] used multiscale temporal convolution and exploited the correlation of the original data to better model the channel topology. Literature [10] describes the skeleton features using Lie groups, then describes the relationship of these features in time order by dynamic temporal regularization, and finally uses multiclass support vector machines for the behavior recognition task. Literature [11] designs a multifeature fusion coding method based on VLAD. Literature [12] designs the spatiotemporal weight coding method based on skeleton features. Literature [13] constructs a motion feature generator based on the existing generative adversarial network framework to perform the learning of judgment optical flow features. Literature [14] investigates temporal pooling and long-term information dependence of behavioral features on the basis of CNNs. In the literature [15], the decomposition model of convolutional networks on spatiotemporal sequences is investigated, i.e., the 3D spatiotemporal convolution is decomposed into 2D spatial convolutional kernel and 1D temporal convolutional layer to accomplish the representation and recognition of human behavior. Literature [16] further investigates the combined strategy of 2D spatial convolution and 1D temporal pooling. Literature [17] extends 2D convolutional operations into 3D convolution and implements a dual-stream I3D. In the literature [18], in order to complete the extraction of human behavior on spatiotemporal features, a dual-stream pooling network is designed to further enhance the feature representation. In the literature [19], a synchronous appearance and relationship module SMART are proposed, and the learning of spatiotemporal features of behavior is accomplished by stacking the model. Literature [20] designs a multi-Fiber network, each Fiber uses lightweight convolution, and the speed of behavior recognition is greatly improved.

In the process of moral education evaluation in colleges and universities, schools can conduct in-depth mining based on big data and provide reference for student management and education service supply by analyzing student classroom behavior data to achieve overall improvement of education level. In order to make full use of the action features in the human skeleton sequence and achieve lightweight action recognition model with improved recognition accuracy, this paper proposes a lightweight adaptive graph convolutional network combining multi-information flow data fusion and spatiotemporal attention mechanism. The human skeleton-based action recognition is very little affected by factors such as illumination and background and has great advantages over the RGB data-based methods. The joint skeleton data of human body are a topological graph, and each joint point in the graph has different number of neighboring joints. Traditional convolutional neural networks cannot directly use the same size convolutional kernel for convolutional computation to process such non-Euclidean data. Therefore, in the field of skeleton-based behavior recognition, a graph convolutional network-based approach is more suitable. The experimental results show that the recognition accuracy of the algorithm in this paper is high, and it can do the work of moral evaluation better.

## 2. Methodology

### 2.1. Student Behavior Algorithm

#### 2.1.1. Graph Convolutional Network

In the Euclidean space represented by an image, each pixel in the image is treated as a node, then the nodes are arranged regularly and the number of neighboring nodes is fixed, and the points on the edges can be padding operation. However, in a non-Euclidean space like the graph structure, the nodes are disordered and the number of neighbor nodes is not fixed, and feature extraction cannot be achieved by a traditional convolutional neural network with a fixed size convolutional kernel. A convolutional kernel capable of handling variable-length neighbor nodes is needed [21]. For the graph, features need to be extracted by inputting a feature matrix *I* of dimension *T*  *×  vF* and an adjacency matrix *G* of *T* *×* *T*, where *T* is the number of nodes in the graph and *F* is the number of input features per node. The nodal feature transformation formula for the adjacent hidden layer is shown below.

(1) $$\begin{array}{c} B^{x+1}=f\left(B^{x},G\right), \end{array}$$

where *x* is the number of layers, the first layer (*B*^0^=*IB*^0^). *f*(·) is the propagation function, and the propagation function varies for different graphical convolutional network models. Each layer *B*^*x*^ corresponds to the *T* × *F*^*x*^-dimensional feature matrix, and the aggregated features are transformed into the features of the next layer by the propagation function  *f*(Δ), which makes the features more and more abstract.

#### 2.1.2. Lightweight Graph Convolutional Network Framework

**Figure 1:**
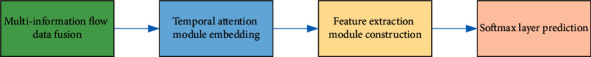
Network framework.

In order to make full use of the action features in human skeleton sequences and to achieve a lightweight action recognition model with improved recognition accuracy, this paper proposes a lightweight adaptive graph convolutional network combining multiple information streams data fusion and spatiotemporal attention mechanism. Taking the input human skeleton sequence as the research object, we first fuse four kinds of data information: joint point information flow, bone length information flow, joint point offset information flow, and bone length change information flow. Then, an embeddable spatiotemporal attention module based on nonlocal operations is constructed to focus on the most action discriminative joints in the human skeleton sequence after the information flow data fusion. Finally, the recognition results of the action fragments are obtained by Softmax, and the main framework of the network is shown in Figure 1.

#### 2.1.3. Multi-Information Flow Data Fusion

At present, the methods based on graph convolution [22] mostly adopt multiple training under a variety of different data sets and carry out decision-making level fusion according to the training results, resulting in a large amount of network parameters. Therefore, the original joint point coordinate data are preprocessed before training to realize the data-level fusion of joint point information flow, bone length information flow, joint point offset information flow, and bone length change information flow, so as to reduce the network parameters and reduce the calculation requirements. The definition of joint points of human skeleton sequence is shown in formula (2).

(2) $$\begin{array}{c} s=\left\{Q_{x,n}/x=1,2,\ldots ,T;n=1,2,\ldots ,N\right\}, \end{array}$$

where *N* is the total number of frames in the sequence, *T* is the total number of nodes18, and *x* is the nodes at the moment  *n*. Before fusing the multiple information streams, a diverse preprocessing of the skeleton sequence *s* is required. The node information stream is obtained from the coordinates of 18 nodes obtained by the human pose estimation algorithm OpenPose, which is a significant cost reduction compared to motion capture devices. Other information streams are defined as follows.

Bone Length Information Flow: the node near the center of gravity of the body is defined as the source node, and the coordinates are used to obtain the bone length information flow by making the difference between the two nodes, as shown in the formula (3).

(3) $$\begin{array}{c} H_{x,y,n}=Q_{y,n}-Q_{x,n}\\ =\left(i_{y,n}-i_{x,n},j_{y,n}-j_{x,n}\right). \end{array}$$

Joint Difference Information Flow: the coordinates of the joint point *x* of the *n*th frame are defined as ( *Q*_*x*,*n*_=(*i*_*x*,*n*_, *j*_*x*,*n*_)), and the coordinates of the joint point *x* of the (*n* + 1)-th frame are expressed as (*Q*_*x*,*n*+1_=(*i*_*x*,n+1_, *j*_*x*,*n*+1_)). The joint difference information Fflow can be obtained by making a difference between the coordinates of the same joint point in adjacent frames, and the formula is shown in formula (4).

(4) $$\begin{array}{c} YD_{x,n,n+1}=Q_{x,n+1}-Q_{x,n}\\ =\left(i_{x,n+1}-i_{x,n},j_{x,n+1}-j_{x,n}\right). \end{array}$$

Change of Bone Length Information Flow: in two adjacent frames, the same section of the bone due to the action changes caused by the different lengths, defined by the formula (3) the *n*th frame of the bone length information flow is  *H*_*x*,*y*,*n*_, then the (*n* + 1)th frame of the bone length information flow is  *H*_*x*,*y*,*n*+1_, by the same bone length of adjacent frames for the difference to obtain the bone length change information flow. The formula is shown in formula (5).

(5) $$\begin{array}{c} CH_{x,n,n+1}=H_{x,y,n+1}-H_{x,y,n}. \end{array}$$

**Figure 2:**
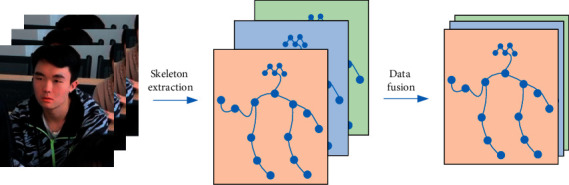
Data fusion of information flow.

As shown in Figure 2, the multiple data streams are weighted and fused into a single feature vector according to the definitions of articulation point information stream, bone length information stream, articulation point offset information stream, and bone length change information stream. The skeleton sequence dimension is changed from (4 × *N* × *Y* × *C*_1_)*Q* to 1 × *N* × *Y* × 4*C*_1_ as shown below.

(6) $$\begin{array}{c} \text{Fusion}=\left\{\omega_{1}Q_{x,n}+\omega_{2}H_{x,y,n}+\omega_{3}YD_{x,n,n+1}+\omega_{4}CH_{x,n,n+1},\;x=1,2,\ldots ,T;n=1,2,\ldots ,N\right\}, \end{array}$$

where the weight *ω*_1_ ~ *ω*_4_ is determined by the joint point offset degree (*σ*_1_(*σ*_1_ ∈ [0° ~ 360°])) and the bone length change degree ( *σ*_2_(*σ*_2_ ∈ [0 ~ 100%])). *σ*_1_ is the angle of the line formed by the coordinate point *Q*_*x*,*n*_ in the previous frame and the coordinate point *Q*_*x*,*n*+1_ in the next frame and the coordinate origin, respectively, and *σ*_2_ is defined as formula (7).

(7) $$\begin{array}{c} \sigma_{2}=\frac{\left|Q_{y,n+1}-Q_{x,n+1}\right|-\left|Q_{y,n}-Q_{x,n}\right|}{\left|Q_{y,n}-Q_{x,n}\right|}, \end{array}$$

where the absolute value operation represents the bone length, when *σ*_1_ ≥ 30° and *σ*_2_ ≤ 50%, *ω*_1_ and *ω*_3_ weights are 2, *ω*_2_ and *ω*_4_ weights are 1.

When *σ*_1_ ≤ 30° and *σ*_2_ ≥ 50%, the weights of *ω*_1_ and *ω*_3_ are 1, and the weights of *ω*_2_ and *ω*_4_ are 2. When *σ*_1_ and *σ*_2_ are less than the threshold, the weights are 1. When both *σ*_1_ and *σ*_2_ are greater than the threshold, the weights are 2. By calculating the offset degree of joint points and the change degree of bone length, higher weight is given to the information flow data with large change degree, so as to enhance the representation of action by information flow. Then the fused single feature vector is used to represent the multi information flow data, and the training times are reduced from 4 times to 1 time, which reduces the amount of overall parameters, so as to improve the network operation speed.

#### 2.1.4. Temporal Attention Module Construction

It is also important to ensure the accuracy of action recognition on the basis of the increased speed of network computing. A human skeleton sequence contains all information in the temporal and spatial domains, but only the nodal association information that is discriminative for some of the actions is worthy of attention. The attention mechanism mostly just removes irrelevant terms and focuses on the action region of interest, and the real redundant information comes from other aspects.

**Figure 3:**
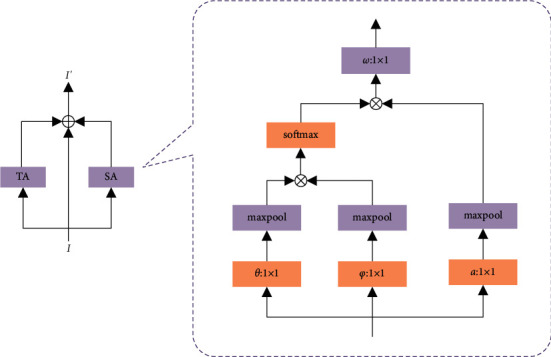
Spatio-temporal attention module.

The joint point with offset degree *σ*_1_ ≥ 30° of each joint point is defined as the source joint point, and one source joint point is selected at a time, while the other joint points are the target joint points. The local operation method in the neural network can only calculate the correlation between two individually after traversing the target nodes, so that the source nodes lose the global characterization ability. In order to characterize the correlation of all target nodes to source nodes, as shown in Figure 3. The idea of nonlocal operations is incorporated into the spatiotemporal attention module, and a max pool layer of size 2 × 2 and step size 2 is added after the feature input to ensure that the number of data and parameters are compressed while preserving the original features as much as possible.

The spatiotemporal attention module (STA) contains a spatial attention module and a temporal attention module. The spatial attention module (SA) captures the intraframe joint correlation, and the temporal attention module (TA) captures the interframe joint correlation, and finally the two are summed and fused with the input features. The output features of the temporal attention module have the same dimension as the input, and thus can be embedded between the network structures of the graph convolutional network.

The implementation of the module features is divided into 4 steps.

(1) The dimension of the infeed feature *I* is  *N* × *T* × *C*, where *T*, *N*, and *C* correspond to the number of frames, joints, and channels, respectively. The input features of the spatial attention module are represented as *k*=[*k*_1_^*s*^, *k*_2_^*s*^,…, *k*_*T*_^*s*^] ∈ *R*^*N*×*T*×*C*^.
(2) Embedding the features into the Gaussian function (*θ* and  *φ*, convolution kernel dimension  1 × 1) calculates the correlation of two joints *i* and *j* at any position, enumerated by  *j*, and obtains the weighting of the joints  *i*, represented as shown below.
  (8) $$\begin{array}{c} j_{x}^{s}=\frac{1}{C\left(k^{s}\right)}\sum_{qy}f\left(k_{x}^{s},k_{y}^{s}\right)a\left(k_{y}^{s}\right), \end{array}$$
where *k*_*x*_^*s*^ and *k*_*y*_^*s*^ denote the features of the nodes *x* and  *y*, respectively. The function *a* is used to calculate the feature representation of the node  *y*, and (*a*(*k*_*y*_^*s*^)=*M*_*a*_^*s*^*k*_*y*_^*s*^)*M*_*a*_^*s*^ is the weight matrix to be learned. The Gaussian function *f* is defined as shown below.
  (9) $$\begin{array}{c} f\left(k_{x}^{s},k_{y}^{s}\right)=e^{\theta {\left(k_{x}^{s}\right)}^{N}\varphi \left(k_{y}^{s}\right)}. \end{array}$$
Where (*θ*(*k*_*x*_^*s*^)=*M*_*θ*_^*s*^*k*_*x*_^*s*^, *φ*(*k*_*y*_^*s*^)=*M*_*φ*_^*s*^*k*_*y*_^*s*^), (*C*(*k*^*s*^)=∑_*qy*_*f*(*k*_*x*_^*s*^, *k*_*y*_^*s*^)) is set as the normalization factor of the correlation representation. In order to reduce the computational cost and maximize the retention of low-order features, a maximum pooling layer of size 2 × 2 and step size 2 is added after the functions *θ*,*φ*, and *a*.
(3) The spatial attention information *o*_*x*_^*s*^(*o*_*x*_^*s*^ ∈ *R*^*N*×*T*×*C*^) is obtained by making the function weighted.
  (10) $$\begin{array}{c} o_{x}^{s}=M_{o}^{s}j_{x}^{s}. \end{array}$$
(4) Denote the infant features of the temporal attention module as *k*^*n*^=[*k*_1_^*n*^, *k*_2_^*n*^,…, *k*_*T*_^*n*^] ∈ *R*^*N*×*T*×*C*^.The temporal attention information *o*_*x*_^*n*^(*o*_*x*_^*n*^ ∈ *R*^*N*×*T*×*C*^) is obtained by repeating (2) and (3), and the temporal attention information *o*_*x*_(*o*_*x*_ ∈ *R*^*N*×*T*×*C*^) is obtained by adding and fusing with the spatial attention information and the infant features.

(11) $$\begin{array}{c} o_{x}=o_{x}^{s}+o_{x}^{n}+k_{x}. \end{array}$$

The discriminative spatiotemporal association information of the nodes is obtained by the attention mechanism based on nonlocal operations, and the interference of irrelevant terms in the action region and the input redundant node information is removed, which reduces unnecessary calculations and thus improves the accuracy.

#### 2.1.5. Spatio-Temporal Feature Extraction Module Construction

In order to extract the features of the skeleton sequence in spatial and temporal dimensions, the dynamic skeleton is first modeled using the spatiotemporal graph convolutional network and a spatial partitioning strategy, and the original expression is shown below.

(12) $$\begin{array}{c} I_{\text{out}}=\sum_{x}^{Z}M_{x}\left(I_{xt}G_{x}\right)⊙W_{x}, \end{array}$$

where *I*_in_ and *I*_out_ are the graph convolutional input and output features, respectively, *Z* is the spatial domain convolutional kernel size, *M*_*x*_ is the weight, *G*_*x*_ is the adjacency matrix of node  *x*, ⊙ represents the dot product, and W_*x*_ is the mapping matrix of nodes given connection weights.

**Figure 4:**
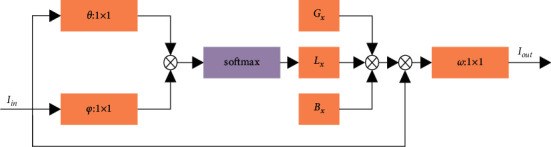
Adaptive graph convolutional module.

Since all mnemonic actions cannot be accurately identified using predefined skeleton structure data, an adaptive adjacency matrix *G*_*x*_ is needed to make the graph convolutional network model adaptive. Therefore, in order to change the topology of the skeleton sequence graph in network learning, the adjacency and mapping matrices that determine the topology in formula (12) are divided into (*G*_*x*_, *B*_*x*_) and *L*_*x*_. The block diagram of the adaptive graph convolution module is shown in Figure 4, and the output features are reconstructed as shown below.

(13) $$\begin{array}{c} I_{\text{out}}=\sum_{x}^{z}M_{x}I_{xt}\left(G_{x}+B_{x}+L_{x}\right). \end{array}$$

In Figure 4, *θ* and *φ* are the Gaussian embedding functions in formula (9), and the convolution kernel size is  1 × 1. The first part *G*_*x*_ is still the adjacency matrix of the node *x*.The second part *B*_*x*_ is an additive complement to the original adjacency matrix, which can be updated iteratively through network training. The third part *L*_*x*_ is continuously driven by the data to learn the connection weights, and the node correlation can be calculated by formula (8) and then multiplied with the 1 × 1 convolution to obtain the similarity matrix *L*_*x*_.

(14) $$\begin{array}{c} L_{x}=\text{soft max}\left(I_{xt}^{N}M_{\theta x}^{N}M_{\varphi x}I_{xt}\right). \end{array}$$

Through the above calculation, the adaptive graph convolution module is constructed, and then the spatiotemporal information contained in the skeleton sequence is extracted.

**Figure 5:**
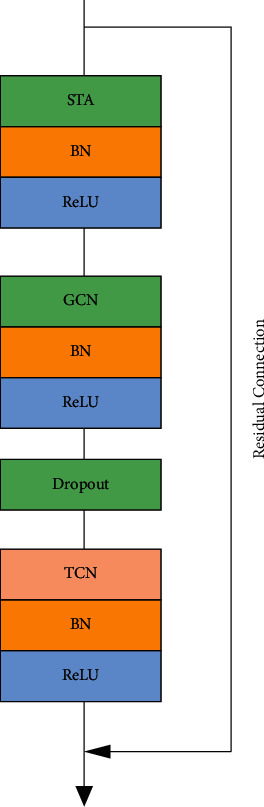
Spatiotemporal feature extracting module.

The spatiotemporal feature extraction module proposed in this paper is shown in Figure 5. The data are normalized by BN (batch normalization) layer after each convolution operation, and then the model expression capability is improved by ReLU layer. The embeddable spatiotemporal attention module STA has been built in Section 2.1.1, and the features are input to the extraction module to extract the action nodes of interest. Then, the correlation of each joint point of the same frame in the skeleton data is obtained in the spatial dimension by the adaptive GCN, and the relationship of the same joint point of adjacent frames is obtained in the temporal dimension by the temporal convolutional network (TCN). The dropout layer reduces the interaction of hidden layer nodes to avoid overfitting of the graphical convolutional network, and the parameter is set to 0.5, while the residual connection is performed to increase the stability of the model.

#### 2.1.6. Overall Network Structure Construction

**Figure 6:**
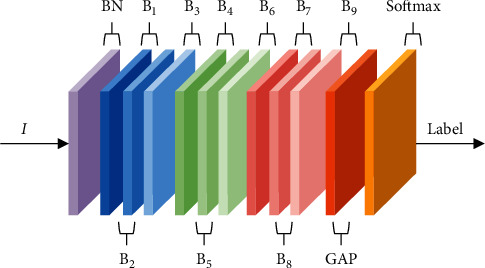
Overall network architecture.

As shown in Figure 6, the nine spatiotemporal feature extraction modules B1∼B9 are stacked. In the direction from feature input I to behavior label output, BN layer is used for normalization after skeleton map input, B1∼B3 output feature dimension is Batch × 64 × *T* × *N*, B4∼B6 output feature dimension is Batch × 128 × *N*/2 × *T*, B7∼B9 output feature dimension is Batch × 256 × *N*/4 × *T*, where the number of channels are 64 The global average pooling (GAP) operation is applied in the spatial and temporal dimensions to unify the feature map sizes of the samples, and finally the data from 0 to 1 are obtained using the Softmax layer for the recognition of human behavior.

### 2.2. Moral Education Evaluation System in Colleges and Universities

#### 2.2.1. Database Design

In the design of moral education quality evaluation system for college students, the database system is an important material basis for carrying out the work related to comprehensive quality evaluation system for college students, and it plays an important role in the system design and application. Scientific design of database can provide efficient ways and technical support for data storage, avoid data redundancy, and also realize data integrity and unity. The corresponding system can be combined with the basic database structure to build an effective input interface and input format to ensure convenient and effective data input and build a complete basic database for comprehensive student quality evaluation.

#### 2.2.2. System Software and Hardware Design

**Figure 7:**
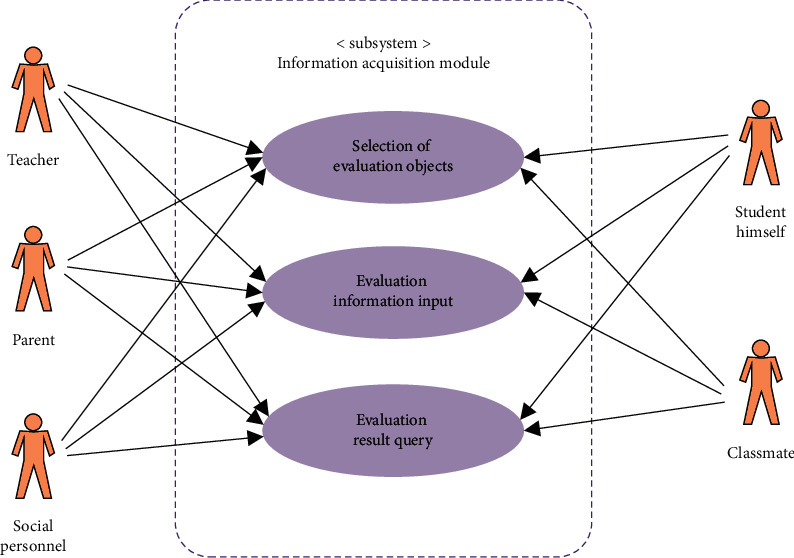
UML use case diagram for information acquisition module.

The hardware design of the moral quality evaluation system for students in colleges and universities should focus on the data collection terminal and data receiving terminal. The specific management software design is the core part of the whole fault management system, which has a direct impact on the system being able to find the data source quickly and accurately in the data information management. In the software design, the data information management program is designed to achieve effective search of data sources, while the simulation program is used to simulate the parameter signals of large electromechanical integration equipment after docking between the management system and the integration equipment to achieve effective data extraction. In this regard, the management software design takes Windows 2000 as the basic software platform, with the help of VC for interface design, and stores the management mode related to the electronic control system through the database management of access to realize the effective system management program design. The sensor, MCU, AD chip, and other components constitute the data acquisition side. The receiving end is also connected by multiple asynchronous serial ports and also connected with LCD, chip, and other components. And the bus is connected to the wireless transmission module, whose function is equivalent to the terminal receiving device, which can transmit the signal to the control center with the help of antenna, and then transmit the received data information to the host location. In the system hardware design, focus on effective control system architecture and good wiring design. And in the system software design, it contains the data acquisition node, coordinator node, and the main controller design. Through the serial port to receive environmental information from the wireless network, do a good job of parsing and processing, and then save the relevant information and transmit it to the GPRS module to receive relevant control commands or other student quality information data with the help of the serial port. The functional design of this system is shown in Figure 7.

## 3. Result Analysis and Discussion

### 3.1. Algorithm Performance Comparison

**Figure 8:**
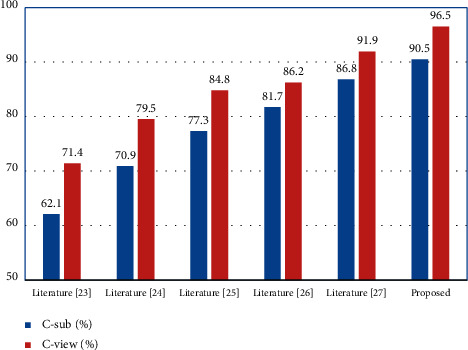
Compared with different algorithm on NTU RGB + D.

**Figure 9:**
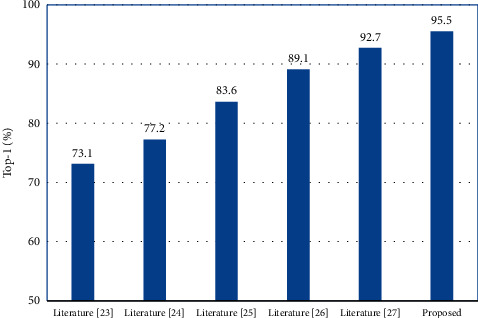
Compared with different algorithm on N-UCLA.

The model performance of this paper's model is compared with those of literature [23–27] on the NTU RGB + D and N-UCLA data sets, as listed in Tables 1 and 2. Also, Table 3 comparison results are presented visually in the form of bar graphs in Figures 8 and 9. The comparison shows that the proposed algorithm of this paper has the best performance.

### 3.2. Analysis of Classroom Behavior Recognition Accuracy

Table 3 lists the classification accuracy rates of 10 types of behaviors commonly seen by teachers and students in the classroom when the model of this paper is used. It can be seen that the classification accuracy rates of most behaviors are over 90%, among which picking up actions and raising hands actions are more easily recognized accurately because of the larger magnitude of the whole body, reaching 98.2% and 98.7% recognition accuracy rates, respectively. For the offending actions (such as playing with the phone), a high recognition rate of 95.3% was also achieved. However, for the recognition of static actions such as writing, although it did not reach the recognition accuracy of other actions, it still had 82.6% recognition accuracy.

## 4. Conclusion

The behavior recognition technology based on big data can effectively analyze the classroom behaviors of teachers and students, provide support for moral education evaluation in colleges and universities, and improve the efficiency and comprehensiveness of moral education evaluation in colleges and universities. In this paper, we propose a lightweight graph convolutional network combining multi-information flow data fusion and spatiotemporal attention mechanism to address the core problem in the field of moral education effect evaluation and student behavior analysis in colleges and universities, namely, the recognition speed and recognition rate of two types of algorithms for convolutional neural networks and graph convolutional networks are not high. By combining multi-information stream data fusion with adaptive graph convolution and also improving feature utilization by embedding spatiotemporal attention module, the performance of the model in this paper is optimal and also the recognition accuracy is improved substantially when tested and compared on NTU RGB + D and N-UCLA data sets. The design and improvement of this system can help universities to better carry out comprehensive student assessment and improve their human education. The follow-up work can make more improvements in two aspects: improving the accuracy of individual action recognition and continuing to propose a more lightweight model.

## Figures and Tables

**Table 1:** Compared with different algorithm on NTU RGB + D.

Algorithm	C-sub/%	C-view/%

Literature [23]	62.1	71.4
Literature [24]	70.9	79.5
Literature [25]	77.3	84.8
Literature [26]	81.7	86.2
Literature [27]	86.8	91.9
Proposed	90.5	96.5

**Table 2:** Compared with different algorithm on N-UCLA.

Algorithm	Top-1/%

Literature [23]	73.1
Literature [24]	77.2
Literature [25]	83.6
Literature [26]	89.1
Literature [27]	92.7
Proposed	95.5

**Table 3:** Classroom action recognition accuracy.

Action	Accuracy (%)

Sit down	97.4
Raise hands	98.7
Play phone	95.3
Check time	96.8
Drink water	93.5
Eat food	90.8
Pick up	98.2
Stand up	97.8
Write	82.6

## Data Availability

The labeled data set used to support the findings of this study is available from the author upon request.
